# Impact of pregabalin treatment on synaptic plasticity and glial reactivity during the course of experimental autoimmune encephalomyelitis

**DOI:** 10.1002/brb3.276

**Published:** 2014-09-02

**Authors:** Gleidy A A Silva, Fernando Pradella, Adriel Moraes, Alessandro Farias, Leonilda M B dos Santos, Alexandre L R de Oliveira

**Affiliations:** 1 Laboratory of Nerve Regeneration, Department of Structural and Functional Biology, Institute of Biology, University of Campinas – UNICAMPCampinas, SP, Brazil; 2 Neuroimmunology Unit, Department of Genetics, Evolution and Bioagents, University of Campinas – UNICAMPCampinas, SP, Brazil; 3 Neuroimmunomodulation Group, Department of Genetics, Evolution and Bioagents, University of Campinas – UNICAMPCampinas, SP, Brazil

**Keywords:** Experimental autoimmune encephalomyelitis, pregabalin, spinal motoneurons, synapse

## Abstract

**Background:**

Multiple sclerosis (MS) is an autoimmune and neurodegenerative disease that affects young adults. It is characterized by generating a chronic demyelinating autoimmune inflammation in the central nervous system. An experimental model for studying MS is the experimental autoimmune encephalomyelitis (EAE), induced by immunization with antigenic proteins from myelin.

**Aims:**

The present study investigated the evolution of EAE in pregabalin treated animals up to the remission phase.

**Methods and results:**

The results demonstrated a delay in the onset of the disease with statistical differences at the 10th and the 16th day after immunization. Additionally, the walking track test (CatWalk) was used to evaluate different parameters related to motor function. Although no difference between groups was obtained for the foot print pressure, the regularity index was improved post treatment, indicating a better motor coordination. The immunohistochemical analysis of putative synapse preservation and glial reactivity revealed that pregabalin treatment improved the overall morphology of the spinal cord. A preservation of circuits was depicted and the glial reaction was downregulated during the course of the disease. qRT-PCR data did not show immunomodulatory effects of pregabalin, indicating that the positive effects were restricted to the CNS environment.

**Conclusions:**

Overall, the present data indicate that pregabalin is efficient for reducing the seriousness of EAE, delaying its course as well as reducing synaptic loss and astroglial reaction.

## Introduction

Multiple sclerosis (MS) is an autoimmune and neurodegenerative disease that affects young adults worldwide. MS usually affects women in the range of 18–40 years old (Bentzen et al. 2010; Sellner et al. 2011; Ahlgren et al. 2012; Mackenzie et al. 2014). The initial symptoms can be diplopia and blurred vision and, with the evolution of the disease, loss of balance, paraplegia and tetraplegia can be observed (Kornek et al. 2000; Olsson et al. 2000; Swanborg 2001). MS may be identified in three different forms: relapsing-remitting, primary progressive and secondary progressive (Bluestein et al. 2012; Kaya et al. 2012; Cadavid et al. 2013). Although the etiology of the disease is not completely understood, the use of immunomodulators has provided improvements to most of the patients. However, the development of new treatment approaches or drug combinations is necessary in order to improve the quality of life of MS patients (Hickey 1999; Achiron and Gurevich 2006).

An experimental model for studying MS is the experimental autoimmune encephalomyelitis (EAE) that is, usually induced in mice and rats (Balashov et al. 1997). EAE can mimic a disease exacerbation followed by remission, or it can be induced in a relapsing-remitting form (Butterfield et al. 1999).

Recent studies have shown the important role of IL-17 in the development of EAE, so that CD4+ T cells, that produce such cytokine, were denominated Th17 lymphocytes. The differentiation of Th17 cells is regulated by a complex network of cytokines, including IL-6 (Bettelli et al. 2006; Korn et al. 2009).

According to Fitzgerald et al. (2007), IL-27 inhibits the differentiation of Th17 cells and its absence increases the differentiation and cell infiltration in the CNS, thus contributing to the exacerbation of EAE.

We have previously shown that important synaptic changes take place in the spinal cord during the exacerbation of the disease, what may involve glutamatergic excitotoxicity (Marques et al. 2009; Scorisa et al. 2011). Also, during peak disease, neurotoxic substances are released, such as nitric oxide and glutamate agonists, both by resident microglia and infiltrating macrophages (Hammarberg et al. 2000). The excess of glutamate causes depolarization of cells with increase in intracellular calcium, which leads to degradation of intracellular elements (Pin and Duvoisin 1995). Such events can contribute directly to the functional deficits observed during disease exacerbation, particularly affecting the nerve terminals (pre-synaptic inputs) in apposition to the cell body of spinal motoneurons (Marques et al. 2006; Freria et al. 2010; Scorisa et al. 2011). It is believed that to a certain extent the motoneurons with less input can focus on the metabolism initially to survive the injury and later to regenerate (Cullheim et al. 2002). During the evolution of degenerative processes in the CNS, over activation of astrocytes and microglia can be harmful to the spinal circuits, what may in turn decrease the motor recovery. In this sense, the use of immunomodulators has proved to reduce astrogliosis and microglial reaction. In turn, the utilization of other neuroprotective substances may enhance the effectiveness of the current drugs used to treat MS. One possibility is the pregabalin, which is a protein with structural analogies with GABA, although it does not bind to GABA receptors. It binds to the *α*2*δ* subunit of voltage-controlled calcium channels. Due to its efficient blockage of such channels, it significantly reduces Ca2+ influx, reducing release of glutamate. In the present study, we have tested whether the use of pregabalin (Silver and Miller 2004; Ha et al. 2008; Rasouli et al. 2009), that attenuates the levels of calcium entering the cytoplasm of cells could be helpful in reducing the seriousness of EAE, delaying its course as well as reducing synaptic loss and astroglial reaction.

## Material and Methods

### Animals

Female adult Lewis rats were obtained from the Multidisciplinary Center for Biological Investigation (CEMIB/UNICAMP). They were kept under 12 h light/dark cycle, receiving food and water ad libitum until 7–8 weeks old and approximately 200 g of body weight. The experiments were developed following the standards of the Brazilian College of Animal Experimentation (COBEA), and were approved by the Committee for Ethics in Animal Use – Institute of Biology – CEUA/IB/UNICAMP, under protocol no. 2287-1.

### Experimental autoimmune encephalomyelitis induction (EAE) and treatement with pregabalin

The rat model was chosen for its reproducibility as well as for the monophasic profile of the disease, which mimics an exacerbation of the illness. The animals were immunized with myelin basic protein (MBP, Sigma, St. Louis, MO; 40 *μ*g/animal) diluted in complete Freund's adjuvant (CFA; Sigma). For greater stimulation of the immune response, an extra amount of Mycobacterium tuberculosis (MT, H37RA, Difco, Detroit, MI, 2.0 mg/mL) was added to CFA. The immunization was carried out by a subcutaneous injection, on both flanks of each animal (Brocke et al. 1996). Animals treated with pregabalin received 30 mg/kg/day of the drug (gavage; Eutamene et al. 2000; Ha et al. 2008, 2011). The first dose was administered immediately after the animals recovered from anesthesia. The treatment was repeated daily until the time the animals were killed. Placebo counterparts received vehicle solution (0.9% phosphate buffered saline).

### Evaluation and grading the progression of EAE

The signs and symptoms of disease were analyzed on a daily basis and were scored according to clinical observations. The degree 0 was considered as absence of clinical signs; 1 – loss of tonus of the tail; 2 – paralysis of one hind limb; 3 – paralysis of both hind limbs, corresponding to the lumbar intumescence demyelination (which occurred approximately by the 12th day after immunization) and remission (starting point of improvement of the clinical signs of disease; Sternberger et al. 1989).

One group was killed during the peak disease (grade 3) and another 10 days after the start of remission.

### Motor evaluation during the course of the disease and treatment with pregabalin

Functional evaluation was performed through the “Walking Track Test” (CatWalk). The animals, prior to immunization, were trained to walk in the equipment in order to obtain the normal gait pattern. All animals have undergone functional evaluation every other day during the course of the disease.

### Euthanasia of animals for immunohistochemistry

The animals were pre-anesthetized with halothane (Tanohalo®, Cristália, Brazil) and then with a mixture of Vetaset (ketamine, Fort Dodge, IA, 50 mg/kg) and Kensol (xylazine Körnig, Argentina, 10 mg/kg). Following thoracotomy, all animals were perfused transcardially with the aid of a peristaltic pump. Initially, aiming at total washing of vessels and organs, the animals were perfused with buffered saline (NaCl 0.9% sodium phosphate buffer, pH 7.38). The fixation was carried out by subsequent perfusion of formalin (10% formaldehyde solution) in sodium phosphate buffer, pH 7.4.

### Processing for immunohistochemical analysis

After fixation, the specimens were dissected and kept in fixative (formalin 10%) for 12 h at 4°C. After this period, the lumbar spinal cords (L4 to L6 segments) were frozen cryoprotected in 30% sacarose and embedded in Tissue-Tek (Miles Inc., Torrance, CA). Histological sections, 12 *μ*m thick, were obtained in a cryostat (Micron HM25). The sections were transferred to gelatinized slides and stored at −20°C until the immunolabeling experiments were carried out.

For immunohistochemistry, the slides were initially acclimatized and immersed in PB 0.1 mol/L, and subsequently incubated in a moist chamber with 100 *μ*L of solution containing 3% BSA for 60 min. The sections were then incubated with primary antibodies diluted in the incubation solution (PB 0.1 mol/L, 1% BSA and 0.3% Triton X100). The primary antibodies were applied and the incubation period was of 4 h. The following primary antibodies were used: goat anti-GFAP (1:1500; Santa Cruz, Dallas, TX), rabbit anti-synaptophysin (1:200; Dako, Glostrup, Denmark) and rabbit anti-IBA-1 (1: 700, Wako, Osaka, Japan). In sequence to the first incubation, the slides were washed in PB 0.1 mol/L and incubated with secondary antibodies (CY-3, Jackson Lab., West Grove, PA) for 60 min. The sections were washed in PB 0.1 mol/L and mounted PBS/glycerol (3:1).

### Quantitative analysis of the immunohistochemistry results

Immunolabeled slides were observed under fluorescence microscope (Nikon Eclipse TS100, Tokyo, Japan) using the appropriate filters for CY3. For each antibody, five representative images from each specimen were obtained at the ventral horn of the spinal cord, using a highly sensitive camera (Nikon, Tokyo, Japan, DXM1200F).

For quantification, the integrated density of pixels that represents the intensity of labeling, was obtained in eight equidistant areas around the motoneurons (synaptophysin immunolabeling) or from the entire picture (IBA-1 and GFAP) using IMAGEJ software (version 1.33 u; National Institutes of Health, Bethesda, MD). The integrated density of pixels was calculated for each animal, and then established the average value for each group ± standard error (SE).

### Preparation of specimens for real-time PCR

The animals were pre-anesthetized (10 days after immunization) with halothane (Tanohalo®, Cristália, Brazil) then with a mixture of Vetaset (ketamine, Fort Dodge, 50 mg/kg) and Kensol (xylazine Körnig, 10 mg/kg). The inguinal and popliteal lymph nodes were removed, macerated and placed in 1 mL of Trizol (Invitrogen, Darmstadt, Germany), being frozen for later extraction of RNA. The RNA extract (1 *μ*g per experimental group) was converted into cDNA using the conversion kit from Applied Biosystems (Foster City, CA). The cDNA was added along with the primers of interest and the appropriate buffer (Master Mix). The analyses were performed in a real-time PCR equipment system (9700; Applied Biosystems), using TaqMan ampliprep.

The primers used herein were specific to IL-6, IL-10, IL-27, TGF*β*, TNF*α*, IL-17 and IFN*γ*. All analyses were performed using *β*-actin as internal control and the normal group as calibrator. The method to quantify PCR data was the 2^−δCT^ technique, according to Livak and Schmittgen (2001).

### Statistical analysis of the results

The results are expressed as mean ± standard error of mean. The intergroup differences analysis was performed by the two-way ANOVA followed by the post-test of Bonferroni. In the case of nonparametric data analysis, the Mann–Whitney post-test was performed. In all analyses, differences between groups were considered to be significant when *P* < 0.05 (*), *P* < 0.01 (**) and *P* < 0.001 (***).

## Results

### Effects of pregabalin treatment on the course of EAE

**Figure 1:**
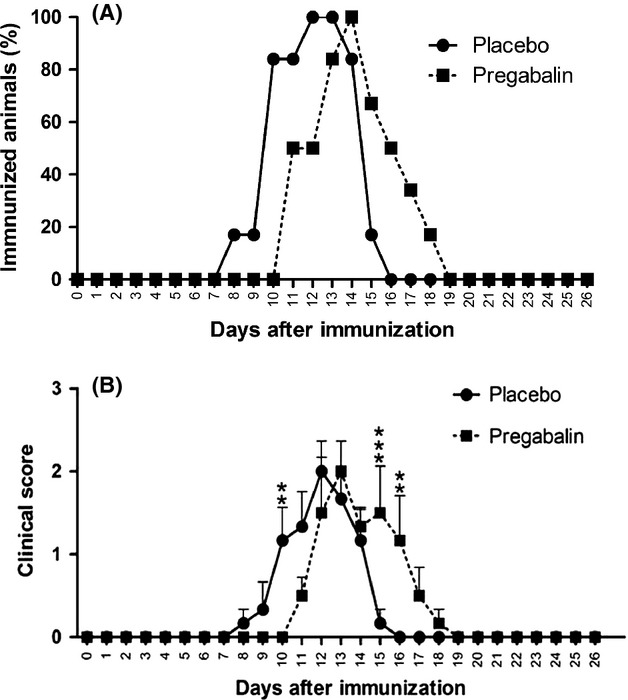
(A) Delayed course of the disease after pregabalin treatment – number of experimental autoimmune encephalomyelitis degree 3 animals. (B) Delay of the appearance of clinical signs of disease, following pregabalin treatment – clinical scores. *N* = 6 for each experimental group.

During the postimmunization period, the animals were evaluated on a daily basis, in the following placebo- and pregabalin-treated groups. The clinical conditions were assessed according to the degree of the disease, to identify the peak disease (grade 3). Figure1A shows the time course of EAE and the percentage of immunized animals that reached degree 3. The results indicate a delay in the onset of clinical signs in the group treated with pregabalin, when compared to the placebo group.

Figure1B represents the average clinical score throughout the experiment in both placebo- and pregabalin-treated animals. The results reinforced a delay in the establishment of the disease, which was statistically significant on day 10 following immunization (*P* < 0.01).

### Motor evaluation during the course of the disease and treatment with pregabalin

**Figure 2:**
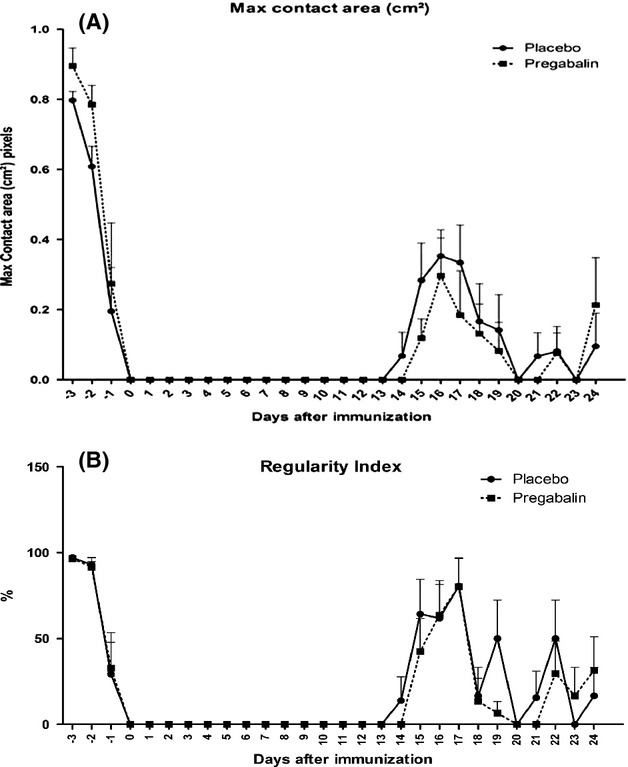
(A) Animals treated with pregabalin showed less prolonged support of hind limbs during walking, indicating better motor coordination. (B) Graph indicating a better regularity of gait in animals treated with pregabalin throughout the course of the disease. *N* = 6 for each experimental group.

For the motor evaluation, the maximum contact area (cm²) of each hind limb footprint (Fig.2A) and the regularity Index (Fig.2B) was obtained with the walking track test (Catwalk System, Noldus, the Netherlands). Both parameters were analyzed with a two-way ANOVA for comparison of treatment and disease evolution time.

Both the maximum area of contact and regularity index showed no significant difference following pregabalin treatment. However, for the regularity index, there is a trend for a positive effect of pregabalin treatment, when evaluating the area under the curve at peak disease.

### Quantitative analysis of the immunohistochemistry

#### Synaptophysin

**Figure 3:**
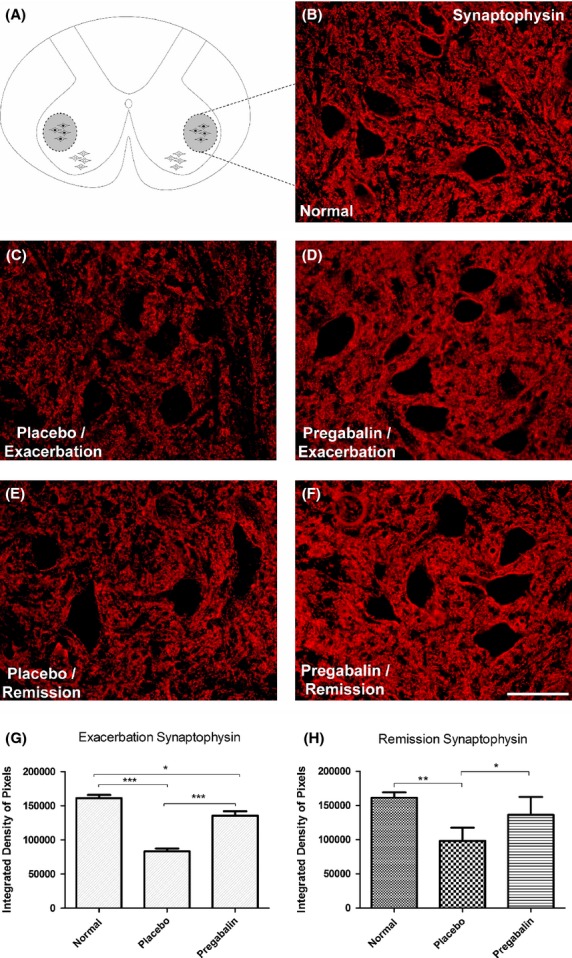
(A) Diagram showing the region of lamina IX where the motoneurons are present. (B) Normal synaptophysin labeling. (C) Loss of synaptophysin immunoreactivity in the placebo group, especially in the neuropil adjacent to motoneurons, evidencing synaptic loss. (D) Evidence of significant synaptic preservation following pregabalin treatment. (E and F) Anti-synaptophysin labeling at the remission phase. Note that there is significant difference between placebo- and pregabalin-treated groups. (G) Quantification of the immunolabeling in the different experimental groups during peak disease. (H) Quantification of the immunolabeling in the different experimental groups during remission phase. *P < 0.05, **P < 0.01, ***P < 0.001. Scale bar 50 *μ*m. *N* = 6 for each experimental group.

Synaptophysin is a protein found in presynaptic vesicles, allowing a general analysis of spinal cord circuit integrity. The immunostaining around the motoneuron cell bodies at lamina IX was analyzed both in placebo- and pregabalin-treated animals. When compared, at the peak of the disease (Fig.3C and D), it is observed that the treatment resulted in significant preservation of spinal cord synaptic circuits, particularly those close to the motoneurons. In the placebo group, it was still possible to observe decreased punctate staining on the surface of the motoneurons, less intensely seen in the pregabalin-treated group. In both cases, there was decrease of immunoreactivity as compared to the normal group with no induction of disease. However, pregabalin treatment was effective in preserving presynaptic terminals in apposition to motoneurons (Normal 1.6 × 10^5^ ± 4.6 × 10^3^; Placebo: 8.3 × 10^4^ ± 4.2 × 10^3^; Pregabalin 1.4 × 10^5^ ± 6.5 × 10^3^).

In the remission phase of the disease (Fig.3 E and F), there was also a significant difference between the control group (normal) and the other groups. Also, significant differences between the placebo- and pregabalin-treated could be observed. (Normal 1.6 × 10^5^ ± 4.6 × 10^3^; Placebo 9.8 × 10^4^ ± 7.9 × 10^3^; Pregabalin 1.4 × 10^5^ ± 1.1 × 10^4^).

#### IBA-1

**Figure 4:**
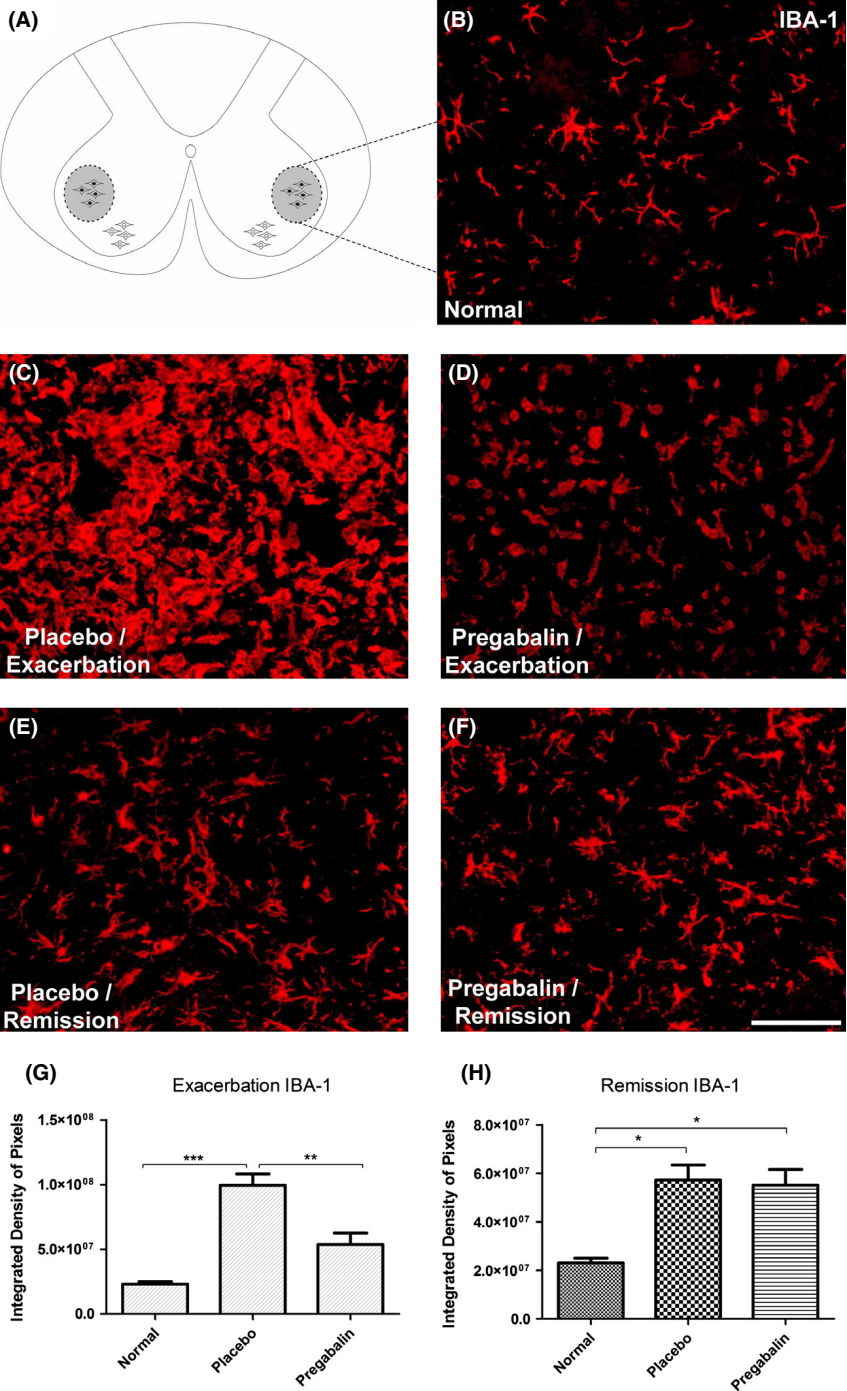
(A) Diagram showing the region of lamina IX where the motoneurons are present. (B) Normal IBA-1 labeling. (C) Increase of IBA-1 immunoreactivity in the placebo group, especially in the neuropil adjacent to motoneurons, during peak disease. (D) Amelioration of the microglial reaction and decrease of monocyte influx following pregabalin treatment. (E and F) Anti-IBA-1 labeling at the remission phase. Note that there is no difference between placebo- and pregabalin-treated groups. (G) Quantification of the immunolabeling in the different experimental groups during peak disease. (H) Quantification of the immunolabeling in the different experimental groups during remission phase. *P < 0.05, **P < 0.01, ***P < 0.001. *N* = 6 for each experimental group. Scale bar 50 *μ*m.

The immunolabeling anti IBA-1 was used to evaluate the microglial reaction as well as the infiltration of macrophages derived from monocytes to the spinal cord microenvironment. Firstly, the morphological aspect of the resident microglia was studied in the control group compared with the placebo- and pregabalin-treated group. In Figure4, the labeling of microglial profiles at lamina IX of Rexed (motor nucleus) revealed a decrease in reactivity in response to the administration of pregabalin, restricted to the exacerbation phase of EAE. Comparing the placebo with the normal control group, a significant increase in the number of activated macrophages during the peak disease could be depicted, clearly showing the moto-neurons surrounded by such cells. Treatment with pregabalin significantly ameliorates such microglial response (Normal 2.30 × 10^7^ ± 1.98 × 10^6^; Placebo 9.9 × 10^7^ ± 8.7 ×10^6^; Pregabalin 5.4 × 10^7^ ± 8.9 × 10^6^, *P* < 0.05).

In the remission of the disease, a general reduction of IBA-1 immunoreactivity could be detected in all groups. Nevertheless, both placebo- and pregabalin-treated animals still showed higher expression of IBA-1, in comparison to the normal control. (Normal 2.3 × 10^7^ ± 1.9 × 10^6^; Placebo 5.7 × 10^7^ ± 6.2 × 10^6^; Pregabalin 5.5 × 10^7^ ± 8.9 × 10^6^, *P* < 0.05).

#### GFAP

The use anti-GFAP antiserum allowed evaluating the astroglial reactivity during the course of the disease.

**Figure 5:**
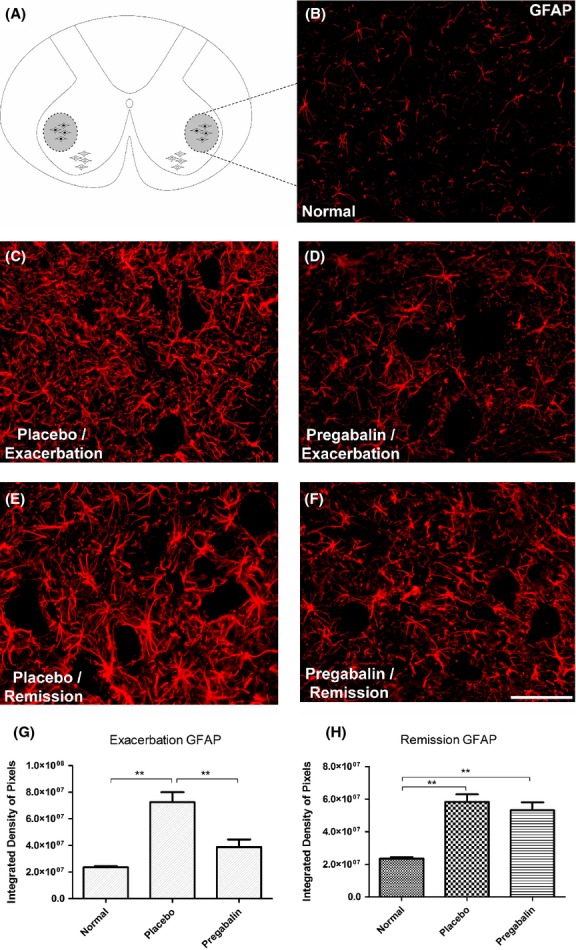
(A) Diagram showing the region of lamina IX where the motoneurons are present. (B) Normal GFAP-1 labeling. (C) Increase of GFAP immunoreactivity in the placebo group, especially in the neuropil adjacent to motoneurons, during peak disease. (D) Amelioration of the astrogliosis following pregabalin treatment. (E and F) Anti-GFAP labeling at the remission phase. Note that there is no difference between placebo- and pregabalin-treated groups. (G) Quantification of the immunolabeling in the different experimental groups during peak disease. (H) Quantification of the immunolabeling in the different experimental groups during remission phase. *P < 0.05, **P < 0.01, ***P < 0.001. *N* = 6 for each experimental group. Scale bar 50 *μ*m.

The analysis of the groups during peak disease demonstrated a significant difference between control and placebo, as well as between placebo and pregabalin treated. Moreover, no statistically significant differences between control and pregabalin treated animals could be observed. Figure5 illustrates that pregabalin treatment is effective in reducing the development of astrogliosis during the peak disease (Normal 2.3 × 10^7^ ± 1.9 × 10^6^; Placebo 5.7 × 10^7^ ± 6.2 × 10^6^; Pregabalin 5.5 × 10^7^ ± 8.9 × 10^6^).

In the phase of remission, however, the pregabalin-treated animals presented an increase in GFAP labeling, which was similar to the placebo-treated group. (Normal 2.4 × 10^7^ ± 8.8 × 10^5^; Placebo 5.8 × 10^7^ ± 4.7 × 10^6^; Pregabalin 5.3 × 10^7^ ± 4.9 × 10^6^).

### Quantitative analysis of cytokines by qRT-PCR

**Figure 6:**
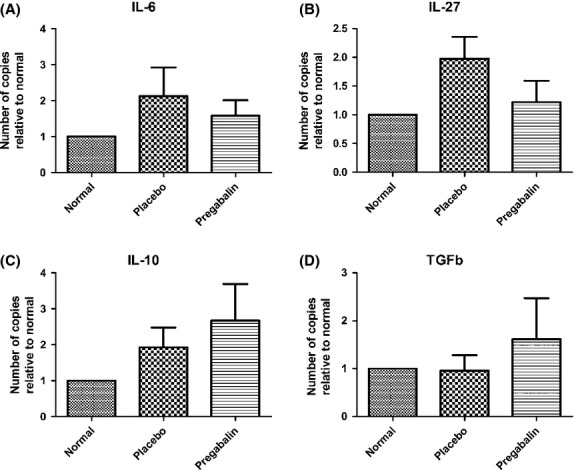
Quantification of the gene transcript levels for IL-6 (A), IL-27 (B), IL-10 (C) and TGFb (D). No statistical differences could be seen among the experimental groups. *N* = 6 for each experimental group.

**Figure 7:**
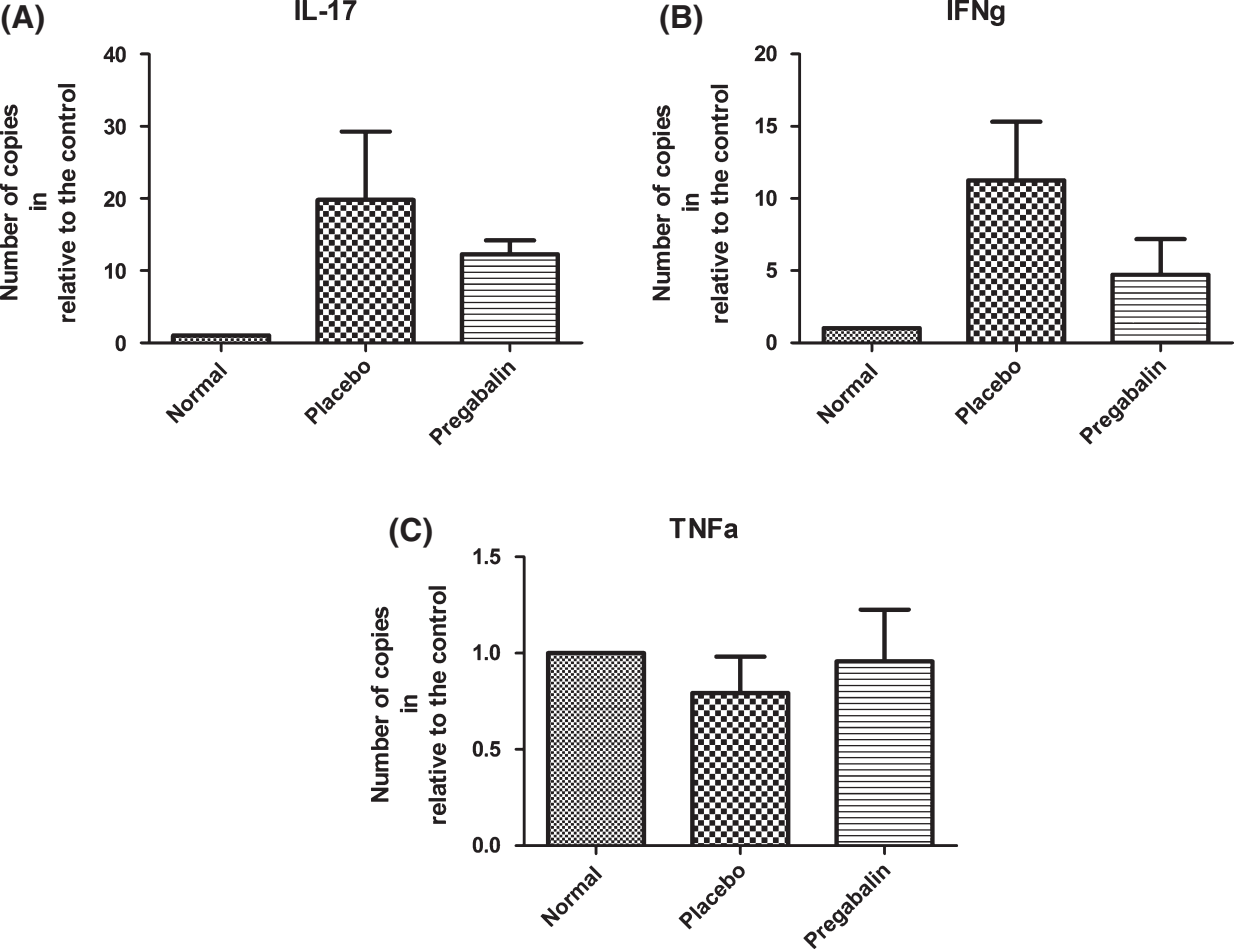
Quantification of the gene transcript levels for IL-17 (A), IFNg (B) and TNFa (C). No statistical differences could be seen among the experimental groups. *N* = 6 for each experimental group.

In the present work, pro-and anti-inflammatory cytokines were analyzed, 10 days after immunization and pregabalin treatment. These particular experiments were carried out with lymph nodes obtained from the inguinal region. Figure6 indicates that pregabalin treatment did not significantly change the levels IL-6, IL-10, IL-27 and TGFb in lymph nodes, indicating a nonimmunomodulatory role of the drug. This trend is reinforced in Figure7 that also show none statistically significant changes in transcript levels of Tnf*α*, IL-17 and IFNg.

## Discussion

Over the years, treatment of multiple sclerosis has been restricted to the use of immunomodulators, such as glatiramer acetate and the recombinant interferon 1b. Such drugs have improved the life quality of patients by retarding the evolution of the disease (Dhib-Jalbut 2003; Blanchette and Neuhaus 2008; Clerico et al. 2008; Jalilian et al. 2012; Goodin 2013). Nevertheless, more efficient treatments are necessary, and the possibility of drug combination could reduce the pace of nervous system deterioration, as a result of subsequent disease exacerbations.

Pregabalin was described as efficient in treating injuries to the spinal cord after mechanical injury (Ha et al. 2008). Such authors have found improvement in functional recovery of treated animals, reduced apoptosis of oligodendrocytes, and decreased activation of microglia. According to Joshi and Taylor (2006), pregabalin reduces the release of neurotransmitters such as GABA, by inhibiting pre-synaptic neurons, without modification of the postsynaptic terminal. This is due to a high binding affinity for the voltage-dependent calcium channels *α*2*δ* of type 1. More recent studies suggest that pregabalin can decrease extracellular glutamate concentration, reducing neuronal death by excitotoxicity (Ha et al. 2011). Overall, the positive effects of pregabalin probably influenced the number of active pre-synaptic terminals to spinal motoneurons, mostly at peak disease. In this way, we believe that such preservation of spinal cord circuits may have important positive influence in the long-term evolution of EAE.

Up to date there are studies associating pregabalin with interferon therapy, in order to treat various types of pain, related to the evolution of MS (Correale 2013). However, there were reports of important side effects related to the use of pregabalin, including delusion (Pollmann and Feneberg 2008; Solaro and Tanganelli 2009). However, such adverse reactions are rare and may probably be overcome with individual dose adjustments.

Taking into account the immunohistochemical results obtained in the present study, pregabalin was efficient during the peak disease, preserving synaptic labeling, as noted by the synaptophysin labeling. Such results were coupled with a significant late appearance of the clinical signs of EAE. Overall, we believe that the synaptic preservation, combined with microglial and astroglial reduced activation contributed to the shortening of time in degree 3 as compared to the placebo group.

Apart from the direct effects of pregabalin in the CNS environment, the present work also investigated whether such a drug could have immunomodulatory properties. For this, lymph nodes from treated animals were evaluated by qRT-PCR to quantitatively access levels of various cytokines. Currently, it is known that Th17 has a key role for the development of immune response. Increased levels of IL-17 correlate with a more severe form of MS, evidenced in normal animals and knockouts (Galicia et al. 2009). Along with the upregulation of Th17, there are other cytokines expressed during the course of EAE, including the IFN-g. This cytokine, acting together with the IL-17, is related to activation of glia in CNS, even before the appearance of clinical signs of disease (Murphy et al. 2010). Additionally, Tnf*α* stimulates inflammatory T cells that will migrate from lymph nodes to the CNS, participating in the process of differentiation of IFNg- and IL-17 producing cells (Gold et al. 2007; Seger et al. 2010). This may in turn affect the process of differentiation of Th17 cells.

Another part of the inflammatory process is related to IL-6 cytokine known to play a key role in the differentiation of Th17 CD4+ cells (Aranami and Yamamura 2008; Murphy et al. 2010). This feature can be demonstrated experimentally by the use of monoclonal antibodies anti-IL-6, which have reduced, mostly, the contingent of Th17 CD4+ cells in the lymph nodes (Aranami and Yamamura 2008; Serada et al. 2008). Our results show that pregabalin was unable to significantly reduce IL-6, indicating that an eventual effect on IL-17 and IFNg may occur as the result of other mechanisms.

In addition to the pro-inflammatory cytokines, the expression of IL-10 was also evaluated in the present study. This molecule has proved to be crucial for increasing the anti-inflammatory response during the course of EAE. In this sense, mice deficient for IL-10 did not show a spontaneous recovery of the disease. It was also observed that mice deficient in IL-10 displayed a greater production of IFNg, demonstrating that this cytokine is directly linked to the regulation of EAE. The present results indicated a trend of IL-10 upregulation following pregabalin administration. Nevertheless, no statistical difference could be obtained.

Taking into account the above-mentioned scenario, the use of pregabalin did not show any significant immunomodulatory effect. In this way, its use may be targeted exclusively to ameliorate the neurological deficits during the course of the disease. The putative combination of pregabalin with glatiramer acetate, for example, could lead to a significant delay in MS evolution, postponing the appearance of the secondary progressive form of the disease. In this regard, further studies in our lab are underway to investigate drug associations, aiming at proposing new strategies to control the evolution of MS, improving the quality of life in patients.
